# Thyroid ultrasound findings in young and middle-aged adults living in the region of the Chornobyl Nuclear Power Plant

**DOI:** 10.1007/s00411-024-01083-2

**Published:** 2024-07-20

**Authors:** Aizhan Zabirova, Alexsey Saiko, Makiko Orita, Fumihiko Furuya, Shunichi Yamashita, Noboru Takamura

**Affiliations:** 1 Department of Global Health, Medicine and Welfare, Atomic Bomb Disease Institute, Nagasaki University Graduate School of Biomedical Sciences, Nagasaki, Japan; 2 Zhitomir Inter-Area Medical Diagnostic Center, Korosten, Ukraine; 3 Department of Thyroid and Endocrinology, Fukushima Medical University, Fukushima, Japan; 4 Global Exchange Center, Advanced Clinical Research Center, Fukushima Medical University, Fukushima, Japan

**Keywords:** Thyroid screening, ^131^I, Ultrasound

## Abstract

Nearly forty years have passed since the Chornobyl Nuclear Power Plant accident, which resulted in childhood and adolescent thyroid cancers increasing due to internal exposure to iodine-131. Therefore, the Fukushima Daiichi Nuclear Power Station accident, in 2011, raised serious anxiety about potential risks of thyroid cancers. Considering the causal relationship between thyroid cancer and the Chornobyl accident, radiation dose to the thyroid due to this accident should be considered carefully. In addition, a thorough investigation of any influence of ultrasound screening of the thyroid on the detection of thyroid diseases was still missing. Consequently, from 2019 to 2021, the frequency of abnormal thyroid findings from screening of residents in Zhytomyr, Ukraine, which was heavily contaminated by the accident, was evaluated in this study. For this, the same diagnostic classification of any thyroid ultrasound findings as those of the Fukushima Health Management Survey were used. This classification used the categories “A1” (no findings), “A2” (thyroid cysts less than 20 mm and/or thyroid nodules less than 5 mm), and “B” (thyroid cysts more than 20 mm and/or thyroid nodules more than 5 mm). 2,978 participants were analyzed. It was found that the frequency of “B” findings increased with age. This may be due to the observed increased incidence of not only malignant but also benign thyroid nodules. It may well be that such an increase will also be observed in Fukushima in the future. It is concluded that future thyroid examiners in Fukushima should be aware of findings specific to adults, such as chronic thyroiditis. For comparison, it will be necessary to perform longitudinal studies in the Japanese population not exposed to radiation from the Fukushima accident.

## Introduction

Nearly forty years have passed since the Chornobyl Nuclear Power Plant (CNPP) accident on 26 April 1986. The main consequence of the exposure of the population to ^131^I released due to the accident was a significant increase of thyroid cancer in children and adolescents, which was correlated to the radiation dose (UNSCEAR 2011). Therefore, in Japan the Fukushima Daiichi Nuclear Power Station (FDNPS) accident of March 11, 2011, raised considerable public concern and serious anxiety about the potential health risks of thyroid cancers. More generally, the so-called “second coming of Chornobyl,” in Fukushima led to such fears worldwide even though the thyroid doses in affected areas of Japan were significantly lower than those in regions affected after the CNPP accident (UNSCEAR 2022).

Consequently, within the framework of the Fukushima Health Management Survey (FHMS), which is being conducted in the Fukushima Prefecture to monitor the overall health conditions of Fukushima residents, those who were less than 18 years old at the time of the Fukushima accident had been evaluated with thyroid ultrasound examinations (Yasumura et al. 2012). Although their dose evaluation suggested relatively low or negligible exposure of ^131^I to the thyroid gland (UNSCEAR 2022), the past 10 years of repeated thyroid ultrasound examinations have revealed more than 200 thyroid cancers among the targeted population of 380,000 individuals (Shimura et al. 2022). Regarding the risk of thyroid cancer among exposed individuals living in the affected areas of the Fukushima Prefecture, only children exposed as infants are predicted to have an increased lifetime risk of thyroid cancer. Therefore, there is a need for close monitoring of this cohort in the future. Despite the debates related to overdiagnosis, the causal relationship between thyroid cancer and the nuclear accident should be carefully considered (Ahn et al. 2014; Togawa et al. 2018). The impact of ultrasound screening of the thyroid on the detection of thyroid disease remains to be clarified. In this context it is important to emphasize that not only results of thyroid dose evaluations but also results of longitudinal ultrasound screening campaigns are relevant.

## Methods

To illuminate the thyroid cancer findings and further contribute to future Fukushima thyroid ultrasound exams, a complementary study of effects of ultrasound thyroid screening was performed in Ukraine. Specifically, from 2019 to 2021, in collaboration with Ukrainian scientists the age-related prevalence of abnormal thyroid findings for residents in the Zhytomyr region of Ukraine, which was heavily contaminated by the Chornobyl accident, was investigated. In the northern area of this region, which is served by the Zhytomyr Inter-Area Medical Diagnostic Center in the city of Korosten, thyroid ultrasound examinations were offered to residents. Out of approximately 40,200 people born between 1977 and 1996 in the area, thyroid ultrasound examinations using 7.75-MHz probes (12 L-RS linear array transducer [GE Healthcare, Japan] and LOGIQ e Expert ultrasound [GE Healthcare, Japan]) were conducted on those who gave consent to participate in this study. Finally, 2,978 participants (709 males and 2,269 females) were screened. Participants were divided into two groups: those born before the accident and exposed to ^131^I (Group 1) and those born after the accident and not exposed to ^131^I (Group 2). In the examinations, the same diagnostic classifications were used as those stipulated in the criteria of the FHMS evaluations in Japan: “A1” (no specific findings), “A2” (thyroid cysts less than 20 mm and/or thyroid nodules less than 5 mm), and “B” (thyroid cysts more than 20 mm and/or thyroid nodules more than 5 mm) (Yasumura et al. 2012). The prevalence of category “B” was assessed by conducting a comparative analysis, while a logistic regression analysis was employed to investigate the potential associations among the prevalence of category “B,” exposure to ^131^I, sex and age at examination. All study protocols were approved by the ethics committee of Nagasaki University Graduate School of Biomedical Sciences (approval no. 20060103-2). Written informed consent was obtained from all participants.

## Results

**Fig. 1:**
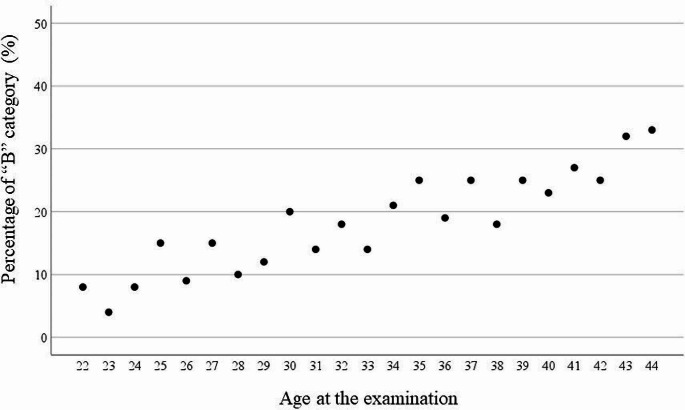
Percentage of “B” category by age at the examination

A total of 2,978 participants were analyzed, of whom 1,743 were born before the accident and belonged to Group 1, while 1,235 were born after the accident and belonged to Group 2. The minimum age in Group 1 was 33 years at the time of the examination, the maximum age was 44 years, and their age at the time of the accident ranged from 1 to 11 years. In Group 2, the age range was 22 to 33 years at the time of the examination. The frequencies of A1, A2 and B in Group 1 were 65%, 11% and 24%, respectively, while the frequencies of A1, A2 and B in Group 2 were 78%, 9% and 13%, respectively. The findings indicate that the frequency of category “B” findings increases with age (Table 1; Fig. 1). Furthermore, the prevalence of category “B” was significantly higher in Group 1 (23.6% vs. 13.3%; *p* < 0.001) than in Group 2. However, regression analysis revealed that this difference vanished after adjusting for age at examination and gender.

**Table 1:** Numbers and percentage of “B” category by age at examination

Age at the examination (y)	Cases of B category (*n*) / Number of subjects (*n*)	Percentage (%)
22	3 / 36	8
23	4 / 94	4
24	7 / 88	8
25	14 / 93	15
26	12 / 128	9
27	18 / 119	15
28	10 /102	10
29	14 / 116	12
30	27 / 137	20
31	15 / 110	14
32	23 / 130	18
33	20 / 140	14
34	33 / 156	21
35	39 / 154	25
36	29 / 151	19
37	37 / 146	25
38	30 / 165	18
39	40 /158	25
40	38 / 166	23
41	48 / 178	27
42	51 / 205	25
43	53 / 167	32
44	13 / 39	33

## Discussion

In this study, the results of thyroid ultrasound examinations of 2,978 individuals currently residing in the Zhytomyr region, who were divided into two age groups were studied: those exposed and not exposed to fallout of the Chornobyl accident in 1986. Because FHMS needed to screen the thyroid glands of a large population of 380,000 children in a manner that would be easy for the public to understand, a simple diagnostic classification (A1, A2 and B) had been developed for the affected population in Japan. In the present study, this classification was adopted for the first time to the affected population in Ukraine. It is noted that cystic and solid lesions were not considered separately, in the present study.

The findings indicate an increase in the frequency of category “B” findings with age among the examined individuals (Table 1; Fig. 1). Specifically, the results obtained suggest that the age-associated increase in category “B” findings may be linked to an elevated frequency of both malignant and benign thyroid nodules. It may well be that such an increase will also be observed in the future in Fukushima prefecture.

This study may have subject selection bias. In particular, the individual thyroid doses of the study participants could not be estimated, as well as other modifying factors such as iodine supplementation status and dietary factors. Consequently, a longitudinal study in a control group of Japanese individuals not exposed to radiation by the accident but adequately matched for ethnicity, age, gender, lifestyle, diet etc. to those exposed individuals already studied by FHMS is needed. Future thyroid ultrasound programs in groups exposed and not exposed by the Fukushima accident should include suspected diagnoses of chronic thyroiditis and adenomatous goiter.

After February 24, 2022, due to the military invasion of Ukraine by the Russian Federation, academic collaborations faced significant challenges in continuing and precisely tracking the diagnoses of abnormal thyroid findings. Nonetheless, longitudinal studies around Chornobyl must be diligently pursued to understand late effects of exposure to relatively high thyroid doses 40 and more years ago.

## Conclusions

In this study, the age-related prevalence of abnormal thyroid findings among a Ukrainian population group was studied by applying a disease classification that had been developed for and applied to individuals in Japan affected by the Fukushima accident. The study results suggest an increase of thyroid cysts more than 20 mm and/or thyroid nodules more than 5 mm with age at examination. In the future, this should be taken into consideration in studies of thyroid cancer prevalence in Japan. It is concluded that longitudinal ultrasound examinations should be conducted by the FHMS in Japan, to take the findings of the present study into account.

## Data Availability

No datasets were generated or analysed during the current study.
